# Efficacy and Safety of Selective Extracranial Radiofrequency Thermocoagulation and Percutaneous Balloon Compression for V3 Trigeminal Neuralgia

**DOI:** 10.1155/prm/3696686

**Published:** 2026-10-06

**Authors:** Hongbo Li, Jiayue Shi, Chenxuan Xiao, Yu Yi, Keyue Xie

**Affiliations:** ^1^ Department of Anesthesiology and Pain Research Center The Affiliated Hospital of Jiaxing University Jiaxing China rzhtcm.com; ^2^ Pinghu Zhimeng Technology Co., Ltd Jiaxing China; ^3^ Department of Anesthesiology Suzhou Xiangcheng People’s Hospital Suzhou China vymaps.com

**Keywords:** percutaneous balloon compression, recurrence rate, selective extracranial radiofrequency thermocoagulation, the proportion of responders, trigeminal neuralgia

## Abstract

**Background:**

Trigeminal neuralgia (TN) is a neuropathic disorder causing paroxysmal severe facial pain. Radiofrequency thermocoagulation (RFTA) and percutaneous balloon compression (PBC) are standard surgical treatments. While RFTA is constrained by its lack of branch selectivity and the risks of repeated intracranial punctures, selective extracranial RFTA (SRFTA) has attracted attention due to its high targeting specificity and fewer neural complications. This study aims to compare the efficacy and safety of SRFTA and PBC in patients with idiopathic TN involving the V3 branch (TN V3).

**Methods:**

This retrospective study included patients with TN V3 who underwent SRFTA or PBC at the First Affiliated Hospital of Jiaxing University between January, 2019 and May 2024. The primary outcome was the proportion of responders at 1 year. Secondary outcomes included the recurrence rate at 1 year, the numeric rating scale (NRS) score at 1 year, and complications such as facial numbness and masticatory muscle weakness.

**Results:**

A total of 236 patients with TN V3 were included. In both the overall cohort and the propensity score‐matched (PSM) cohort, there were no statistically significant differences between the PBC and SRFTA groups in the proportion of responders (overall: 72.7% vs. 77.4%, *p* = 0.499; PSM: 74.5% vs. 77.5%, *p* = 0.743), recurrence rate (overall: 19.0% vs. 18.3%, *p* = 1.000; PSM: 17.6% vs. 17.6%, *p* = 1.000), and incidence of facial numbness (overall: 62.0% vs. 69.6%, *p* = 0.276; PSM: 61.8% vs. 70.6%, *p* = 0.236). However, the incidence of masticatory muscle weakness was significantly higher in the SRFTA group than the PBC group (overall: 12.2% vs. 2.5%, *p* = 0.009; PSM: 11.8% vs. 2.0%, *p* = 0.013). Results from the inverse probability of treatment weighting (IPTW) analysis and subgroup analyses were consistent.

**Conclusion:**

Within the 1‐year follow‐up period, both SRFTA and PBC appeared to be effective therapeutic options for patients with TN V3. However, SRFTA was associated with a higher risk of masticatory muscle weakness. Therefore, PBC may be considered preferentially when minimizing motor complications is clinically important.

## 1. Introduction

Trigeminal neuralgia (TN) is a neuropathic disorder characterized by paroxysmal, drastic stabbing pain in the facial region, typically confined to the trigeminal region. The annual incidence is approximately 4–13 per 100,000 population, with a higher incidence in females than in males, and is most prevalent in people over 50 years of age [1]. The pathogenesis remains incompletely understood, but it is generally believed to involve demyelination of the trigeminal nerve leading to ectopic impulse generation [2]. The intense pain can severely impair the quality of life of patients, leading to anxiety, depression, and even suicidal ideation, thereby imposing a substantial burden on both their physical and mental well‐being.

The trigeminal nerve arises from the trigeminal ganglion (Gasserian) and divides into the ophthalmic (V1), maxillary (V2), and mandibular (V3) nerves, which exit the skull via the superior orbital fissure, foramen rotundum (FR), and foramen ovale (FO), respectively. A systematic review indicates that V3 is most frequently involved in TN, with right‐sided facial involvement more common than left [3]. Currently, treatments for TN are diverse, with pharmacotherapy recommended as the first‐line approach. Although 94% of patients received pharmacotherapy, two‐thirds still experienced moderate to severe pain [4]. Approximately 50% of patients ultimately exhibit a poor response to or intolerance of pharmacotherapy and thus require surgical intervention [5].

TN is categorized into classical, secondary, and idiopathic. Classical TN is characterized by morphological changes of the trigeminal root due to neurovascular compression. Secondary TN arises from an identifiable structural or systemic lesion affecting the trigeminal nerve. Idiopathic TN occurs without demonstrable neurovascular contact or root abnormalities. Microvascular decompression (MVD) is the preferred surgical approach for classical TN, whereas clinical guidelines recommend percutaneous neuroablative procedures for idiopathic TN patients who respond poorly to pharmacotherapy [6].

Radiofrequency thermocoagulation (RFTA) is a routinely employed percutaneous neuroablative procedure in clinical practice. RFTA employs the thermal effect of electrical currents to induce nerve injury, thereby blocking the transmission of nociceptive signals. Although conventional RFTA achieves effective treatment response, consistently achieving precise branch specificity can be clinically challenging [7]. Moreover, repeated intracranial procedures carry the risk of cerebrospinal fluid leakage, intracranial infection, cranial nerve palsy, and intracranial hemorrhage. To enhance targeting specificity and reduce complications, selective extracranial RFTA (SRFTA) has gradually attracted the attention of scholars in recent years [8–12]. Under CT guidance, SRFTA offers high selectivity and preserves the structure and function of nontargeted trigeminal branches to the greatest extent. Previous retrospective studies have reported immediate postoperative response rates exceeding 90% with SRFTA. Furthermore, SRFTA can be performed extracranially, further reducing the risk of intracranial puncture‐related complications. Therefore, SRFTA may be a safe and effective surgical approach for patients with idiopathic TN involving the V3 branch (TN V3). Percutaneous balloon compression (PBC) is another widely used intervention that aims at inflating a balloon within the Meckel’s cave, thereby mechanically compressing the trigeminal ganglion and blocking nerve conduction. This selectively disrupts aberrant nociceptive signal transmission and yields immediate postoperative response rate exceeding 80% [13]. Studies suggest that PBC predominantly damages large myelinated fibers while sparing small fibers, such as those mediating the corneal reflex. The procedure is relatively straightforward and relies primarily on image guidance for localization, resulting in a short learning curve and operative time. It is a relatively simple, well‐established option for idiopathic TN.

Although the two interventions are widely employed, their efficacy and safety in patients with TN V3 remain unclear. Therefore, this study aims to compare SRFTA and PBC in terms of postoperative treatment response, recurrence rate, and related complications in patients with TN V3, thereby offering evidence‐based guidance for clinicians to individualize their choice of intervention according to branch involvement, anatomical considerations, and complication risk.

## 2. Methods

### 2.1. Setting and Study Population

This retrospective study included patients with TN V3 who underwent SRFTA or PBC at the First Affiliated Hospital of Jiaxing University, Zhejiang, between January 1, 2019, and May 17, 2024. According to the third edition of the International Classification of Headache Disorders (ICHD‐3), all participants met the diagnostic criteria for idiopathic TN [14]. This study was reviewed and approved by the Ethics Committee of the First Affiliated Hospital of Jiaxing University (approval NO. 2025‐LP‐603) and was conducted in accordance with the Declaration of Helsinki. As this was a retrospective study, patient informed consent was waived.

### 2.2. Patient Eligibility

Inclusion criteria were (1) age > 18 years; (2) involvement of the V3 branch of TN; (3) disease duration ≥ 3 months; and (4) failure to achieve adequate treatment response or to tolerate drug‐related adverse effects following pharmacotherapy.

Exclusion criteria were (1) incomplete medical records and (2) missing follow‐up data or follow‐up duration < 12 months.

### 2.3. Procedures

#### 2.3.1. Selective Extracranial RFTA

Patients fasted for 4–6 h preoperatively. After establishing intravenous access, they were placed supine on the CT table with a pillow under the shoulders, head tilted approximately 20° and secured with wide adhesive tape. Lead shields were placed over the eyes, neck, and chest. Oxygen was administered via a nasal cannula, and vital signs were continuously monitored. A CT scan in the semi‐coronal plane with 3 mm slice thickness was used to identify the layer with the largest transverse diameter of the FO and to measure the distance and puncture angle from the target (midpoint of the FO internal opening) to the skin surface. Following local anesthesia with 2 mL of 1% lidocaine at the puncture point, a radiofrequency needle was inserted under CT guidance along the planned trajectory to the target position. The stylet was removed, and the radiofrequency generator (Baylis PMG230) was connected to deliver high‐frequency (50 Hz) and low‐frequency (2 Hz) currents for the sensation and motor stimulation test. After confirming accurate RF needle placement and the absence of nontarget branch twitching, deep sedation was administered, and two cycles of continuous ablation at 90°C–95°C for 120 s each were performed. If a 0.5 mA current failed to induce paresthesia or rhythmic twitching in the mandibular region, the needle tip position was adjusted and retested until satisfactory results were obtained.

#### 2.3.2. Balloon Compression Procedure

Under CT guidance, the position of the FO was confirmed, and the insertion trajectory was planned. Local anesthesia was achieved with 1% lidocaine at the puncture point, after which the balloon catheter was introduced through the puncture needle into the FO and advanced into Meckel’s cave. After confirming the position via CT, 0.35–0.70 mL of contrast medium was slowly injected into the balloon until a pear‐shaped configuration was observed radiographically. Following 3–5 min of balloon compression, the contrast medium was aspirated, the catheter was removed, and the puncture site was dressed.

Throughout the procedure, the vital signs of patients were continuously monitored, and appropriate interventions were performed. If blood pressure increased by more than 20% from baseline, urapidil 12.5 mg was administered intravenously to maintain hemodynamic stability. If the heart rate fell below 50 bpm, atropine 0.5 mg was given, and the procedure was temporarily suspended until the heart rate returned to normal. In the event of a subcutaneous hematoma at the puncture site, compression hemostasis was applied, followed by postoperative cold compresses.

### 2.4. Endpoints

The primary outcome was the proportion of responders at 1 year, defined as a reduction in the numeric rating scale (NRS) score of at least 50% from baseline [15–17]. Secondary outcomes included the 1‐year postoperative recurrence rate, the NRS score at 1 year, and complications including facial numbness and masticatory muscle weakness. Recurrence of TN was defined as an increase in the NRS score to > 3 on the operated side after an initial treatment response [18, 19].

### 2.5. Statistical Analyses

This study conducted data analysis using R software (version 4.2.0). Continuous variables with a normal distribution are reported as mean ± standard deviation (SD) and compared by *t*‐test; nonnormally distributed variables are presented as median (interquartile range [IQR]) and compared by Mann–Whitney U test. Categorical variables are expressed as counts (percentages) and compared using the chi‐square test or Fisher’s exact test, as appropriate. Propensity scores (PS) were estimated via multivariate logistic regression, with the model incorporating baseline variables. These included age, sex, BMI, hypertension, diabetes, laterality, disease duration, and preoperative NRS scores. These variables were selected based on clinical relevance and the availability of retrospective data. However, certain clinical factors—such as imaging findings and detailed pain phenotypes—were not systematically recorded in the study dataset and were therefore excluded from the PS model. A 1:1 nearest‐neighbor matching was implemented with a caliper of 0.2 SD of the logit of PS. Post‐matching balance was evaluated by standardized mean differences (SMD), with SMD < 0.1 indicating acceptable balance. After matching, paired McNemar tests were used for categorical outcomes, and paired *t*‐tests or Wilcoxon signed‐rank tests for continuous outcomes. To assess robustness, inverse probability of treatment weighting (IPTW) was applied to the full cohort to re‐establish covariate balance, followed by baseline and outcome analyses in the weighted sample. All tests were two‐sided, and *p* < 0.05 was considered statistically significant.

Building on these analyses, additional exploratory analyses were conducted in the full cohort. First, subgroup comparisons of the primary endpoints—the proportion of responders and recurrence rate at one year—were performed according to predefined stratification factors (gender, age, preoperative NRS score, and disease duration). Second, for each primary endpoint, multivariable regression models adjusting for surgical approach and all baseline covariates were developed to assess the independent effect of surgical approach on the proportion of responders and recurrence rate at 1 year.

## 3. Results

From January 2019 to May 2024, 273 patients with TN V3 underwent SRFTA or PBC, of whom 4 had incomplete medical records and 33 were excluded due to loss of follow‐up. Finally, 236 patients were included in the analysis, of whom 121 were in the PBC group and 115 in the SRFTA group. The flowchart of patient enrollment is summarized in Figure 1. The surgical success diagrams of the SRFTA group and the PBC group are shown in Figures 2 and 3, respectively.

**FIGURE 1:**
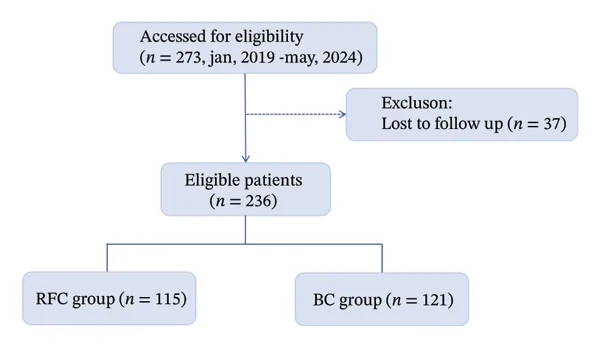
Flowchart of the study.

**FIGURE 2:**
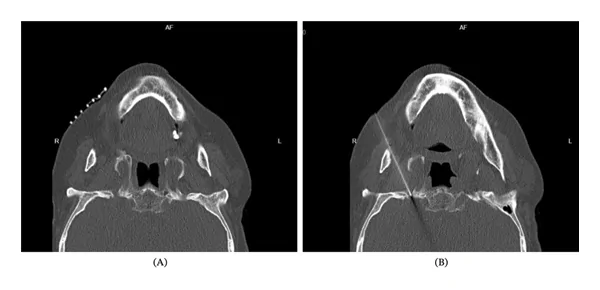
(A) Preoperative CT scan showing the anatomical structure of the foramen ovale. (B) CT scan showing the path and position of the needle located in the right foramen ovale.

**FIGURE 3:**
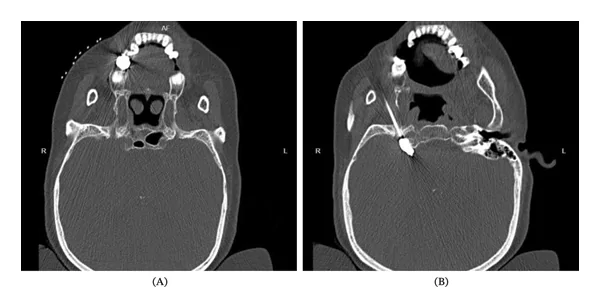
(A) Preoperative CT scan showing the anatomical structure of the foramen ovale. (B) CT images of the balloon after it was filled with iohexol contrast agent.

### 3.1. Baseline Characteristics

In the full cohort, there were no significant differences in baseline characteristics such as age, gender, body mass index, disease duration, hypertension, and diabetes mellitus between groups (*p* > 0.05; Table 1). However, slight imbalance was noted for disease duration (SMD = 0.365), preoperative pain score (SMD = 0.162), and age (SMD = 0.104), all of which exceeded the prespecified SMD threshold of 0.10. After PS matching (PSM), each group comprised 102 patients, and all baseline covariates achieved balance with SMDs below 0.10 (Table 1, Figure 4).

**TABLE 1:** Comparisons of baseline characteristics between PBC and SRFTA before and after propensity score matching.

Characteristic		Total cohort				PSM cohort			
		PBC (*n* = 121)	SRFTA (*n* = 115)	SMD	*p* value	PBC (*n* = 102)	SRFTA (*n* = 102)	SMD	*p* value
Gender, *n* (%)	Male	40 (33.1)	50 (43.5)	0.100	0.13	36 (35.3)	40 (39.2)	0.039	0.664
	Female	81 (66.9)	65 (56.5)			66 (64.7)	62 (60.8)		

Age, (years)		70.00 (63.00, 77.00)	69.00 (60.00, 74.50)	0.104	0.843	70.00 (64.00, 77.00)	68.50 (60.25, 75.00)	0.026	0.649

BMI, (kg/m^2^)		23.15 (21.08, 25.10)	23.44 (21.40, 25.51)	0.092	0.577	23.28 (22.03, 25.62)	23.48 (21.35, 25.95)	0.036	0.743

Hypertension, *n* (%)	YES	48 (39.7)	52 (45.2)	0.060	0.465	41 (40.2)	44 (43.1)	0.029	0.776

Diabetes mellitus, *n* (%)	YES	13 (10.8)	15 (13.0)	0.024	0.748	12 (11.8)	12 (11.8)	< 0.001	> 0.999

Laterality, *n* (%)	Left	45 (37.2)	38 (33.0)	0.048	0.596	34 (33.3)	34 (33.3)	< 0.001	> 0.999
	Right	76 (62.8)	77 (67.0)			68 (66.7)	68 (66.7)		

Disease duration (months)		48.00 (12.00, 108.00)	36.00 (12.00, 84.00)	0.365	0.252	36.00 (12.00, 81.00)	48.00 (12.00, 84.00)	0.036	0.378

Preoperative NRS score		5.00 (5.00, 5.00)	5.00 (5.00, 5.00)	0.162	0.225	5.00 (5.00, 5.00)	5.00 (5.00, 5.00)	0.056	0.75

*Note:* Data are presented as median (IQR) or *n* (%). SRFTA, selective extracranial radiofrequency thermocoagulation.

Abbreviations: BMI, body mass index; NRS, numeric rating scales; PBC, percutaneous balloon compression; PSM, propensity score‐matched.

**FIGURE 4:**
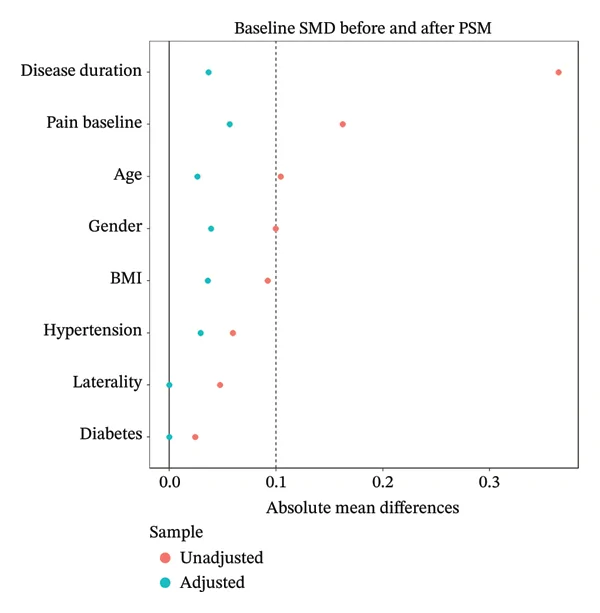
Covariate balance between SRFTA and PBC before and after propensity score matching.

### 3.2. Procedure Effects

In the overall cohort, the proportion of responders at 1 year was 72.7% in the PBC group and 77.4% in the SRFTA group, with no statistically significant difference between the two groups (*p* = 0.499; Table 2). The NRS pain scores at 1 year were 0 (0.3) and 0 (0.2) in the PBC and SRFTA groups, respectively, with no significant difference (*p* = 0.925). The recurrence rates of the PBC group and the SRFTA group were 19.0% and 18.3%, respectively, with no significant difference (*p* = 1.000). After PSM, the proportion of responders at 1 year was 74.5% in the PBC group and 77.5% in the SRFTA group, with no significant difference (*p* = 0.743; Table 2). The median NRS score at 1 year postoperatively was 0 in both groups (*p* = 0.571) and both groups had an identical recurrence rate at 1 year of 17.6% (*p* = 1.000).

**TABLE 2:** Comparisons of outcomes between PBC and SRFTA before and after propensity score matching.

Characteristic		Total cohort			PSM cohort		
		PBC (*n* = 121)	SRFTA (*n* = 115)	*p* value	PBC (*n* = 102)	SRFTA (*n* = 102)	*p* value
Proportion of responders at 1 year, *n* (%)	YES	88 (72.7)	89 (77.4)	0.499	76 (74.5)	79 (77.5)	0.743
NRS score at 1 year		0.00 (0.00, 3.00)	0.00 (0.00, 2.00)	0.925	0.00 (0.00, 3.00)	0.00 (0.00, 2.00)	0.571
Recurrence rate at 1 year, *n* (%)	YES	23 (19.0)	21 (18.3)	> 0.999	18 (17.6)	18 (17.6)	> 0.999
Facial numbness, *n* (%)	YES	75 (62.0)	80 (69.6)	0.276	63 (61.8)	72 (70.6)	0.236
Masticatory muscle weakness, *n* (%)	YES	3 (2.5)	14 (12.2)	0.009^∗^	2 (2.0)	12 (11.8)	0.013^∗^
corneal hypoesthesia, *n* (%)	YES	0 (0)	0 (0)	1	0 (0)	0 (0)	1
Diplopia, *n* (%)	YES	0 (0)	0 (0)	1	0 (0)	0 (0)	1
Hearing impairment, *n* (%)	YES	0 (0)	0 (0)	1	0 (0)	0 (0)	1
Infection, *n* (%)	YES	0 (0)	0 (0)	1	0 (0)	0 (0)	1
Meningitis, *n* (%)	YES	0 (0)	0 (0)	1	0 (0)	0 (0)	1

*Note:* Data are presented as median (IQR) or *n* (%). SRFTA, selective extracranial radiofrequency thermocoagulation.

Abbreviations: NRS, numeric rating scales; PBC, percutaneous balloon compression; PSM, propensity score matching.

^∗^Significant values.

### 3.3. Postoperative Complications

In the full cohort, facial numbness occurred in 62.0% of PBC patients and 69.6% of SRFTA patients (*p* = 0.276; Table 2). However, the incidence of masticatory muscle weakness was significantly higher in the SRFTA group than in the PBC group (12.2% vs. 2.5%, *p* = 0.009). After PSM, the incidence of facial numbness remained nonsignificantly different (61.8% vs. 70.6%, *p* = 0.236), whereas masticatory muscle weakness continued to be significantly more frequent in the SRFTA group than in the PBC group (11.8% vs. 2.0%, *p* = 0.013). No other serious complications, such as corneal hypoesthesia, diplopia, hearing impairment, infection, and meningitis, were observed in either group.

### 3.4. Sensitivity Analysis

To assess the robustness of these findings, we performed an IPTW analysis (Table 3). After weighting, the SMD values of all baseline variables were lower than 0.02. The results of IPTW were consistent with the PSM analysis. There were no significant differences in the proportion of responders at 1 year (77% vs. 71.3%, *p* = 0.346), NRS score at 1 year (0 vs. 0, *p* = 0.855), recurrence rate at 1 year (17.7% vs. 19.8%, *p* = 0.704), and incidence of facial numbness (69.9% vs. 58.9%, *p* = 0.095) between the two groups. The incidence of masticatory muscle weakness in the SRFTA group was still significantly higher than that in the PBC group after weighting (12.4% vs. 1.5%, *p* = 0.001).

**TABLE 3:** Comparisons of baseline characteristics and outcomes between PBC and SRFTA after inverse probability of treatment weighting.

Characteristic		IPTW cohort			
		PBC	SRFTA	*p* value	SMD
*Baseline characteristics*					
Gender, *n* (%)	Male	42.1	42.5	0.954	0.008
	Female	57.9	57.5		

Age, (years)		70.00 (64.00, 77.00)	69.00 (60.00, 74.75)	0.51	0.002

BMI, (kg/m^2^)		23.15 (21.53, 25.38)	23.41 (21.35, 25.51)	0.904	0.015

Hypertension, *n* (%)	YES	45.1	45.1	0.999	< 0.001

Diabetes mellitus, *n* (%)	YES	13.4	13.3	0.977	0.004

Laterality, *n* (%)	Left	33.1	32.7	0.955	0.008
	Right	66.9	67.3		

Disease duration, (months)		36.00 (12.00, 72.00)	36.00 (12.00, 84.00)	0.641	0.017

Preoperative NRS		5.00 (5.00, 5.00)	5.00 (5.00, 5.00)	0.843	0.007

*Outcomes*					
Proportion of responders at 1 year, *n* (%)	YES	71.3	77.0	0.346	0.130

NRS score at 1 year		0.00 (0.00, 3.00)	0.00 (0.00, 2.00)	0.855	0.040

Recurrence rate at 1 year, *n* (%)	YES	19.8	17.7	0.704	0.053

Facial numbness, *n* (%)	YES	58.9	69.9	0.095	0.233

Masticatory muscle weakness, *n* (%)	YES	1.5	12.4	0.001^∗^	0.441

*Note:* Data are presented as median (IQR) or *n* (%). SRFTA, selective extracranial radiofrequency thermocoagulation.

Abbreviations: BMI, body mass index; IPTW, inverse probability of treatment weighting; NRS, numeric rating scales; PBC, percutaneous balloon compression.

^∗^Significant values.

### 3.5. Exploratory Analysis

Subgroup analyses were conducted with the proportion of responders (Figure 5) and recurrence rate at 1 year (Figure 6) as endpoints. Overall, compared with SRFTA, PBC was associated with an OR of 0.71 (95% CI 0.37–1.35; *p* = 0.299) for the proportion of responders at 1 year and an OR of 1.10 (95% CI 0.55–2.19; *p* = 0.782) for recurrence rate at 1 year. Further stratification by gender, age, preoperative NRS score, and disease duration revealed that the odds ratios in each subgroup approximated 1.0, with 95% CIs crossing 1.0 and *p* values ≥ 0.05. These results indicate no statistically significant differences between the SRFTA and PBC groups in the proportion of responders or recurrence rates at 1 year across patient subgroups.

**FIGURE 5:**
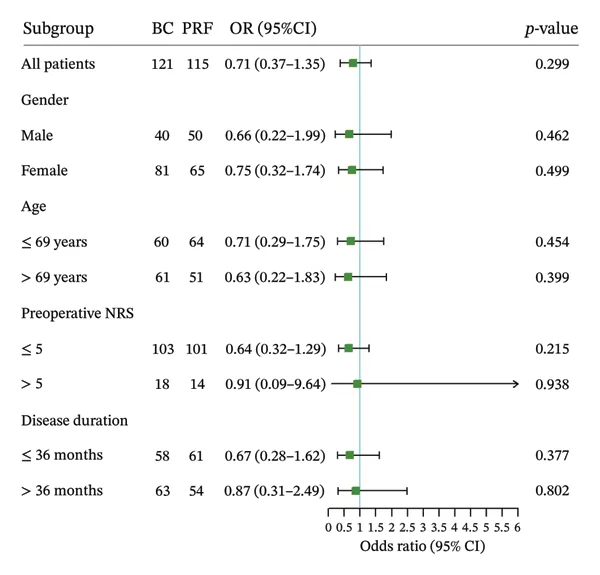
Comparison of the proportion of responders at 1 year between SRFTA and PBC across different subgroups.

**FIGURE 6:**
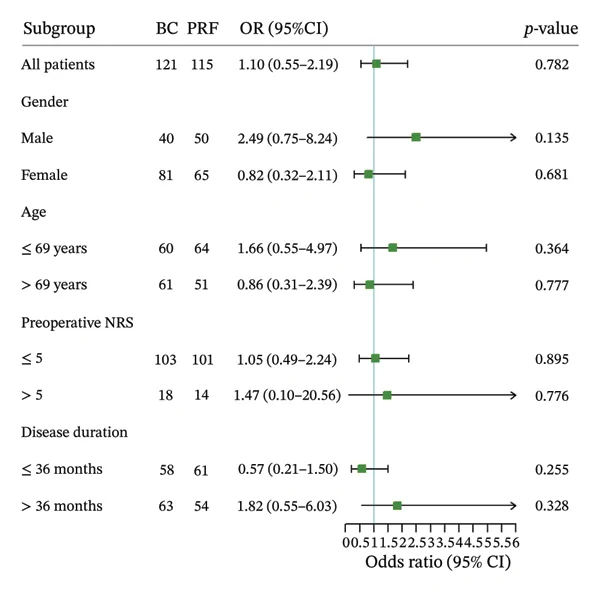
Comparison of the recurrence rate at 1 year between SRFTA and PBC across different subgroups.

After adjusting for baseline covariates, multivariable logistic regression revealed no significant effect of surgical approach on the proportion of responders (OR 0.712, 95% CI 0.372–1.348; *p* = 0.299) and recurrence rate at 1 year (OR 1.102, 95% CI 0.554–2.202; *p* = 0.782) (Table S1).

## 4. Discussion

This study enrolled patients with TN V3 to compare the efficacy and safety of SRFTA and PBC. The results demonstrated a comparable proportion of responders and recurrence rate between the two procedures within the 1‐year follow‐up period, whereas the SRFTA group exhibited a significantly higher incidence of masticatory muscle weakness compared to the PBC group.

This study compared the clinical efficacy of SRFTA and PBC, finding no statistically significant differences in the proportion of responders and recurrence rate at 1 year between the two groups. These findings not only confirm the efficacy of SRFTA for TN V3 but also align with previous studies demonstrating that RFTA and PBC are equally effective. A retrospective study involving 436 patients reported that the postoperative treatment response rates of both RFTA and PBC were approximately 84%, and there was no significant difference in recurrence‐free survival between the two groups [13]. Similarly, in a 24‐month follow‐up study, Jain et al. reported no significant differences in NRS score or recurrence rate between the two groups [20]. Both studies support the comparability of RFTA and PBC in terms of postoperative treatment response. A meta‐analysis of 16 studies revealed that there were no significant differences in postoperative treatment response between RFTA and PBC immediately postoperatively, at 1 month, or at 3 months [21]. However, at 12 months postoperatively, the PBC group exhibited a significantly higher rate of treatment response than the RFTA group. This discrepancy may be attributed to differences in RFTA techniques and patient inclusion criteria. Conventional RFTA typically employs temperatures of 70°C–75°C to selectively destroy small‐diameter, unmyelinated Aδ/C nociceptive fibers through thermal coagulation while relatively preserving large, myelinated Aα/Aβ mechanoreceptive fibers [22]. However, if the temperature is set too low or the thermal damage area is insufficient, nociceptive fibers may persist, leading to incomplete postoperative treatment response or higher recurrence risk. In contrast, SRFTA often uses higher temperatures (90°C–95°C) within a safe and controllable margin to achieve more extensive neural ablation. High‐temperature RFTA can ablate nociceptive fibers more comprehensively, enhancing lesion completeness, which may underlie the improved postoperative treatment response, observed in the SRFTA group [11]. Variations in RFTA parameters across different medical centres may impact long‐term efficacy, highlighting the need for further investigation to elucidate the effect of procedural parameters on long‐term efficacy. This study suggests that the proportion of responders at 1 year in the SRFTA group was not inferior to that in the PBC group among patients with TN V3. Larger prospective studies are needed to validate the impact of different surgical parameters on long‐term outcomes.

Although SRFTA and PBC demonstrate comparable efficacy, the incidence of complications differs between the two cohorts. In our study, the proportion of patients with masticatory muscle weakness in the SRFTA group was significantly higher than the PBC group, which is consistent with the anatomical characteristics of the trigeminal nerve. The motor fibers of the TN pass below the Gasserian ganglion and join the sensory fibers of the mandibular nerve at the FO to exit the skull [23, 24]. Therefore, any intervention targeting the V3 branch will inevitably affect these accompanying motor fibers. SRFTA ablates the nociceptive fibers of the V3 branch via thermal coagulation. However, due to its inherent lack of selectivity among nerve branches, SRFTA renders all fibers within the thermal lesion zone—including both sensory and motor fibers—vulnerable to injury, which leads to their degeneration and necessitates prolonged axonal regeneration. A large cohort study of 137 patients who underwent RFTA showed that approximately 8% of patients experienced masticatory muscle weakness after RFTA, of which 96% had involvement of the V3 branch [25]. The incidence of masticatory muscle weakness reported in previous RFTA studies ranged from 3% to 29%, which is consistent with the 12% incidence in the SRFTA group in our study [26–29]. In contrast, PBC preferentially compresses larger myelinated fibers in Meckel’s cave, transiently impairing conduction without permanent axonal disruption. Previous studies have shown that the incidence of masticatory muscle weakness after PBC is approximately 1%–50%, and most patients can recover spontaneously within a few weeks to months after surgery [21, 30]. In this study, the incidence of masticatory muscle weakness in the PBC group was significantly lower than that in the SRFTA group, while a meta‐analysis by Liu et al. reported that the incidence of masticatory muscle weakness in the PBC group was higher than that in the RFTA group [21]. We speculate that this difference may be due to two aspects. First, SRFTA is usually performed at a higher temperature (90°C–95°C), which has a stronger nerve damage effect and is likely to damage the thick nerve fibers (Aα motor fibers), thereby increasing the risk of masticatory muscle weakness [11]. Second, the meta‐analysis by Liu et al. included all patients with TN, while this study only focused on patients with TN V3, who are more likely to have masticatory muscle dysfunction.

Facial numbness is a common postoperative complication of both SRFTA and PBC. In our study, more than 50% of patients in both groups experienced facial hypoesthesia 1 year after surgery. Tang et al. reported in an elderly cohort that patients who experience postoperative facial numbness had a significantly higher long‐term postoperative treatment response rate than those without numbness (68.1% vs. 55.7%), suggesting that moderate hypoesthesia reflects effective ablation of Aδ and C fibers and thereby indirectly verifies the comparability of the postoperative treatment response observed in our study [25].

### 4.1. Limitations

This study also has several limitations. First, the retrospective, single‐center design makes it difficult to completely exclude confounding factors, including treatment selection bias and attrition bias, or to systematically record certain rare complications such as anesthesia dolorosa, although no severe cases were reported. The choice between SRFTA and PBC may have been influenced by surgeon preference, anatomical factors, and other insufficiently documented variables. Multicenter, prospective randomized controlled trials are warranted to further validate our findings. Second, follow‐up assessments were conducted only at predefined time points, potentially overlooking the evolution of pain and complications at different stages after surgery. Future studies should incorporate more frequent follow‐up intervals to comprehensively evaluate the temporal dynamics of treatment efficacy. Third, the lack of stratification based on the specific nerve branches involved may have influenced the evaluation of clinical outcomes. Future prospective studies should employ stratification or subgroup analysis based on the specific nerve branches involved. Fourth, BNI pain intensity scores and BNI facial numbness scores were not collected in this study, which may limit the standardized assessment of pain and numbness severity. Finally, while we focused on treatment response and common adverse events, we did not assess patient quality of life. Subsequent studies can introduce corresponding scales (such as SF‐36 and EQ‐5D) to improve the efficacy evaluation.

## 5. Conclusions

Within the 1‐year follow‐up period, both SRFTA and PBC appeared to be effective therapeutic options for patients with TN V3. However, SRFTA was associated with a higher risk of masticatory muscle weakness. Therefore, PBC may be considered preferentially when minimizing motor complications is clinically important.

## Author Contributions

Hongbo Li, Jiayue Shi, and Chenxuan Xiao contributed to the design of the clinical trial and supervised the data collection and follow‐up processes. Yu Yi participated in the statistical analysis of the study and drafting this manuscript. Keyue Xie reviews articles and provides surgical support.

## Funding

The authors have no funding sources to disclose.

## Disclosure

All named authors meet the International Committee of Medical Journal Editors (ICMJE) criteria for authorship for this article, take responsibility for the integrity of the work as a whole, and have given their approval for this version to be published. All authors read and approved the final manuscript.

## Ethics Statement

This study was approved by the Ethics Committee of the First Affiliated Hospital of Jiaxing University, located in Jiaxing City, Zhejiang Province, China (2025‐LP‐603). The study was performed in accordance with the Helsinki Declaration of 1964 and its later amendments. Written informed consent was obtained from the individuals for the publication of potentially identifiable images included in Figures 2 and 3.

## Conflicts of Interest

The authors declare no conflicts of interest.

## Supporting Information

Additional supporting information can be found online in the Supporting Information section.

## Supporting information


**Supporting Information** Table S1. Association between procedures and outcomes with logistic regression.

## Data Availability

Clinical data are subject to privacy restrictions and available on reasonable request to the corresponding authors.
